# Pleural Fluid Soluble Interleukin-2 Receptor as a Biomarker for the Diagnosis of Tuberculosis Pleural Effusion: A Systematic Review and Meta-Analysis

**DOI:** 10.1155/2022/4348063

**Published:** 2022-03-20

**Authors:** Zhi Yan, Hua Wang, Wen-Qi Zheng, Zhi-De Hu

**Affiliations:** ^1^Department of Parasitology, The Basic Medical Sciences College of Inner Mongolia Medical University, Hohhot 010050, China; ^2^Department of Laboratory Medicine, The Affiliated Hospital of Inner Mongolia Medical University, Hohhot 010050, China

## Abstract

**Background:**

Several studies have assessed the diagnostic accuracy of pleural fluid soluble interleukin-2 receptor (sIL-2R) for tuberculous pleural effusion (TPE) but with varied results. Therefore, we conducted this systematic review and meta-analysis to evaluate the accuracy of sIL-2R for TPE.

**Methods:**

PubMed, Ovid, and Web of Science databases were searched from inception to 23 March 2021 to identify eligible studies concerning the diagnostic accuracy of fluid sIL-2R for TPE. The sensitivity and specificity of sIL-2R for TPE were pooled with a bivariate model. We estimated the global diagnostic accuracy of PE sIL-2R with a summary receiver operating characteristic (sROC) curve. The revised Quality Assessment for Diagnostic Accuracy Studies tool (QUADAS-2) was used to assess the quality of eligible studies.

**Results:**

A total of nine studies with 270 TPEs and 586 non-TPEs were included in the final analysis. The pooled sensitivity and specificity were 0.81 (95% CI: 0.76–0.86) and 0.92 (95% CI: 0.77–0.98), respectively. The area under the sROC curve (AUC) was 0.82 (95% CI: 0.79–0.86). No significant publication bias was observed.

**Conclusions:**

Pleural fluid sIL-2R is a useful diagnostic marker for TPE. However, the diagnostic accuracies of already available biomarkers such as pleural fluid adenosine deaminase, interferon-*γ*, and interleukin-27 appear to be superior relative to sIL-2R. Therefore, it might not be preferable to use sIL-2R for diagnosing TPE.

## 1. Introduction

Tuberculosis pleural effusion (TPE) is a common cause of pleural effusion (PE) [1], and its diagnosis is a challenge for clinicians. The gold standard for diagnosing TPE is *Mycobacterium tuberculosis* (*Mtb*) culture from pleural fluid or pleural tissue on Ziehl–Neelsen staining [2]. However, the *Mtb* culture is time- and labor-consuming, which did not allow rapid diagnosis. In addition, the sensitivity of *Mtb* culture is less than 20% [3]. Ziehl–Neelsen staining is a rapid diagnostic tool with high specificity, but its sensitivity is less than 5% [3]. Imaging-guided or thoracoscopic pleural biopsy is an effective diagnostic method with high specificity, but its sensitivity is approximately 70% [4]. In addition, biopsy is an invasive procedure and can be associated with complication [5], and its diagnostic accuracy is greatly affected by the operator and observer. Nucleic acid amplification tests (NAATs) are promising molecular diagnostic tools for TPE [6]. Xpert MTB/RIF is the most widely-used commercial NAAT in clinical practice with high specificity. However, its sensitivity is approximately 50% [7]. The low sensitivity may be due to the lack of *Mtb* in pleural fluid [7, 8]. Soluble pleural fluid biomarkers represent another promising diagnostic tool for TPE diagnosis. There are some pleural fluid biomarkers such as adenosine deaminase (ADA), interferon-gamma (INF-*γ*), and interleukin-27 (IL-27) [9]. However, meta-analysis evidence shows that their sensitivities and specificities are around 0.90 [10–12]. Therefore, it remains essential to develop novel pleural fluid diagnostic markers for rapid diagnosis of TPE.

Interleukin-2 (IL-2) is a growth factor that promotes T lymphocyte proliferation in an autocrine manner [13]. Its effect on helper T-cells is mainly mediated by binding to an IL-2 receptor protein (IL-2R). Some studies have shown that IL-2R can be released from the cell surface in a soluble form, termed soluble IL-2 receptor (sIL-2R) [14]. Helper T-cells are a dominant cell population in TPE [15]. These cells can be recruited into the pleural space and involved in the protective immunity against *Mtb* [15, 16]. Several studies have revealed that pleural fluid sIL-2R could be used for the diagnostics of TPE, but the results varied. Herein, we conducted a systematic review and meta-analysis to evaluate the accuracy of sIL-2R for the diagnosis of TPE.

## 2. Methods

### 2.1. Search Strategy

We searched the PubMed, Ovid, and Web of Science databases to identify eligible studies since inception to 23 March 2021. The search algorithm in the PubMed database was (“Tuberculosis, Pleural” (mesh) or “tuberculosis pleural effusion” or “tuberculosis pleurisy” or “tuberculosis pleuritis” or “tuberculous pleurisy” or “tuberculous pleuritis” or “tuberculous pleural effusion” or “pleural effusion^*∗*^” or “pleural fluid^*∗*^”) and (“Receptors, Interleukin-2” (mesh) or “soluble interleukin-2 receptor^*∗*^” or “soluble interleukin-2 receptor^*∗*^” OR sIL-2R OR “Receptors, Interleukin-2” (nm)). We used a similar strategy in searching the Web of Science database. In addition, all references listed in eligible studies were also manually searched. This manuscript is reported by following the PRISMA-DTA (Preferred Reporting Items for a Systematic Review and Meta-Analysis of Diagnostic Test Accuracy Studies) guidelines, which is recommended by the Cochrane Collaboration [17].

### 2.2. Study Selection

All retrieved studies were imported into Endnote to remove duplicate publications. In addition, the studies investigating the diagnostic accuracy of sIL-2R for TPE were included. The exclusion criteria were (i) animal studies; (ii) non-English studies; (iii) conference abstracts, literature review, editorials, and commentary; (iv) studies without sensitivity and specificity data; and (v) if a two-by-two table could not be constructed for meta-analysis. All retrieved studies were independently screened by two reviewers, and any discrepancies were resolved by consensus and full-text reviewing. There is no restriction regarding country, patients age, race, gender, and publication date.

### 2.3. Data Extraction and Quality Assessment

Data extraction was performed by two reviewers independently. The data extracted were name of the first author, country, publication year, components of control, sample sizes of TPE and non-TPE, type of data collection (prospective or retrospective), sIL-2R assay, the reference standard for TPE diagnosis, sensitivity, specificity, area under the receiver operating characteristic (ROC) curve (AUC), and the corresponding cutoff. We constructed a two-by-two table for each eligible study with the sensitivity, specificity, and sample sizes of TPE and non-TPE. The two-by-two table contained the numbers of true positive (TP), false positive (FP), false negative (FN), and true negative (TN).

The revised Quality Assessment for Diagnostic Accuracy Studies tool (QUADAS-2) was used to assess the quality of eligible studies [18].

### 2.4. Statistical Analysis

We used a bivariate model to pool the sensitivity and specificity [19]. Summary ROC (sROC) curve, a global metric of a diagnostic test's accuracy, was constructed to estimate the diagnostic accuracy of sIL-2R [20]. The inconsistency index (*I*^2^) was used to detect potential heterogeneity across eligible studies [21]. The Deeks's test and funnel plot were used to estimate the degree of publication bias [22]. The Stata 13.0 (Stata Corp LP, College Station, TX, USA) with the *midas* command was used for all statistical analyses [23].

## 3. Results

### 3.1. Study Selection Process and Summary of the Eligible Studies

**Figure 1:**
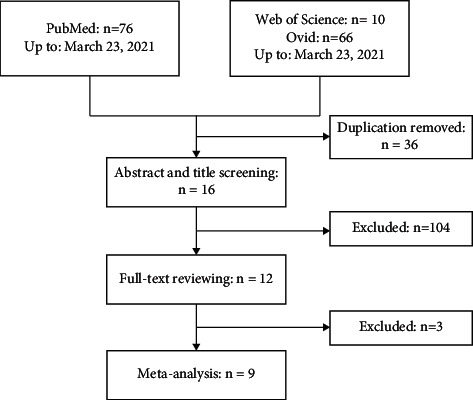
Flowchart of the study selection.

Figure 1 is a flowchart of study selection. Finally, twelve studies met the inclusion criteria, but three did not report sensitivity and specificity [24–26]. Therefore, nine studies [27–35] with 270 TPE and 586 non-TPE patients were included in the present meta-analysis. The summary of the eligible studies is noted in Table 1. Two of the included studies were from China [32, 33], two were from Japan [30, 35], and each of the remaining five studies were from Turkey [27], Poland [28], India [29], Spain [31], and Greece [34]. Sample sizes of the eligible studies ranged from 38 to 173 patients. The disease profile of non-TPE was various, including malignant pleural effusion (MPE) [27–35], parapneumonic pleural effusion (PPE) [27, 28, 30–33, 35], transudative pleural effusion (TRPE) [28, 31–35], heart failure (HF) [30], and uremic pleural effusion (UPE) [32]. Prospective data collection was adopted in three studies [28, 31, 33]. Eight studies determine pleural fluid sIL-2R with ELISA [27–29, 31–35], while one study used diagnostic products corporation (DPC) Immulyze [30]. Culture and biopsy was used as reference standards in all studies, and Ziehl–Neelsen staining was also used in five studies [27–30, 32]. In addition, treatment response was used as a reference standard in five studies [28, 30, 31, 34, 35].

### 3.2. Quality Assessment

Quality assessment of the included studies is shown in Table 2. The patient selection domain in two studies was labeled as high because of inappropriate exclusions [27, 34]. The index domain of three eligible studies was labeled at a high risk of bias because the diagnostic cut-offs were not prespecified [27, 34, 35]. The reference standard domain of all eligible studies was labeled as low. All except two of the eligible studies did not receive the same reference standard or made inappropriate exclusions; therefore, the flow and timing domain of these studies was labeled as high risk of bias [28–32, 34, 35].

### 3.3. Diagnostic Accuracy

The diagnostic accuracy of sIL-2R in eligible studies is summarized in Table 3. Only three studies reported the ROC curve. In three studies, the AUC of sIL-2R was 0.57 [28], 0.80 [29], and 0.96 [31], respectively. Diagnostic cut-off adopted by the eligible studies ranged from 2980 U/mL to 5000 U/mL. The eligible studies' sensitivity ranged from 0.62 to 0.91, and specificity ranged from 0.41 to 1.00.

**Figure 2:**
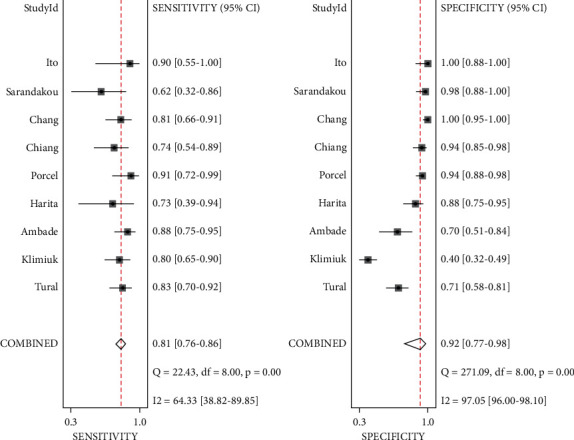
Forest plot of sensitivity and specificity for PE sIL-2R in the diagnosis of TPE.

Figure 2 shows a forest plot of sensitivity and specificity for sIL-2R for the diagnosis of TPE. Pooled sensitivity and specificity were 0.81 (95% CI: 0.76–0.86) and 0.92 (95% CI: 0.77–0.98), respectively. Positive likelihood ratio (PLR) and negative likelihood ratio (NLR) were 10.5 (95% CI: 3.2–34.3) and 0.20 (95% CI: 0.16–0.27), respectively. The diagnostic odds ratio (DOR) was 51 (95% CI: 14–189). The *I*^2^ for sensitivity and specificity was 64% and 97.5%, respectively.

Table 4 lists the positive and negative predictive values (PPVs and NPVs) of sIL-2R for TPE with an assumed prevalence of TPE in the target population. In the target population with the prevalence of TPE <5% in undiagnosed PE, the NPV of sIL-2R was 98.92%. While in the target population with the TPE prevalence >50%, the PPV of sIL-2R was 95.94%.

Because significant heterogeneity was observed in meta-analysis, we analyzed the possible source of heterogeneity among included studies. We hypothesized that sample size (>100 vs. <100), data collection (prospective design vs. retrospective design or unknown), whether the participants were consecutively enrolled (consecutive vs. unknown and non-consecutive), whether treatment response was used to diagnose TPE (yes vs. no) are the potential sources of heterogeneity.  Table 5 lists the results of meta-regression. Although these design characteristics are possible sources of heterogeneity for sensitivity, they lost significance in the joint model. Therefore, the current evidence does not support these design characteristics as sources of heterogeneity.

**Figure 3:**
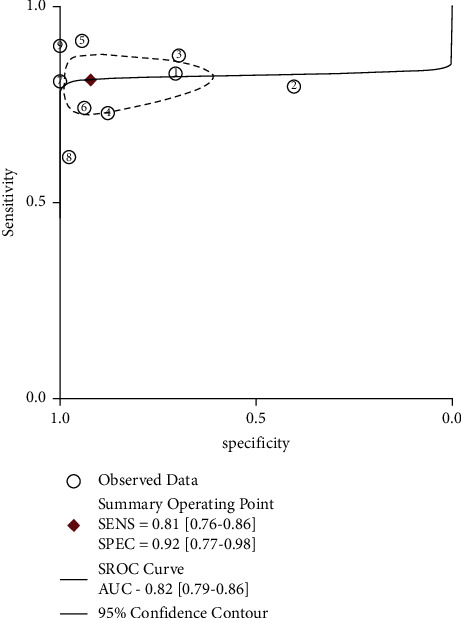
SROC curve for sIL-2R in pleural effusion. SENS, sensitivity; SPEC, specificity; AUC, area under the ROC curve.

The sROC curve of PE sIL-2R is shown in Figure 3. The area under the sROC curve was 0.82 (95% CI: 0.79–0.86).

### 3.4. Publication Bias

**Figure 4:**
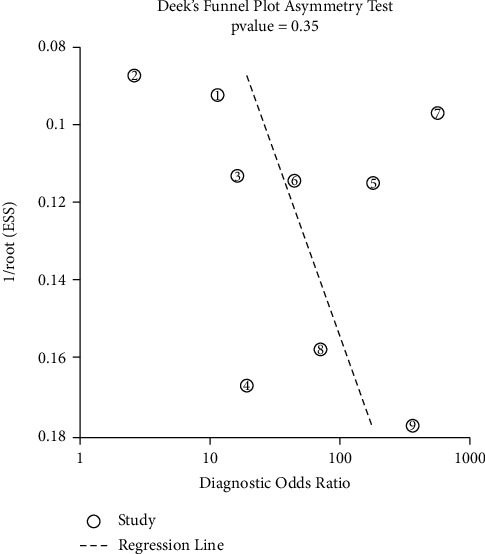
The funnel plot assessment of potential publication bias in studies of sIL-2R in pleural effusion.

The funnel plot indicated that publication bias was not statistically significant (*P*=0.35, Figure 4).

## 4. Discussion

This is the first systematic review and meta-analysis estimating the diagnostic accuracy of PE sIL-2R for TPE to the best of our knowledge. We included nine studies with 270 TPEs and 586 non-TPEs and found that the sensitivity and specificity of PE sIL-2R were 0.81 and 0.92, respectively. The sROC's AUC of PE sIL-2R was 0.82. In addition, no significant publication bias was observed. These results indicate that pleural fluid sIL-2R has a relatively high diagnostic accuracy for TPE.

Sensitivity and specificity are two basic metrics of a diagnostic tool [36]. The pooled sensitivity and specificity of PE sIL-2R were 0.81 (95% CI: 0.76–0.86) and 0.92 (95% CI: 0.77–0.98), respectively. These results mean that 81% of TPE patients had elevated PE sIL-2R and 92% of non-TPE had decreased PE sIL-2R. Therefore, approximately 19% TPE patients will be missed, and 8% of non-TPE patients will be misdiagnosed as TPE if PE sIL-R level was used alone.

A weakness of sensitivity and specificity is that they can only reflect the accuracy of diagnosis at a specific cut-off; therefore, both of them are not a global measure of diagnostic accuracy [36, 37]. By contrast, the AUC of sROC is cut-off independent and thus is a good measure to evaluate the accuracy of an index test [38]. In the traditional ROC curve, each point represents sensitivity and specificity at a certain cut-off. While in the sROC curve, each point represents the data of a single study; therefore, the AUC of sROC represents a globe measure of diagnostic accuracy [39]. The AUC of such curve ranged from 0.5 to 1.0, and a higher value means higher accuracy [20]. We found that the AUC of the sROC curve was 0.82 (95% CI: 0.79–0.86), indicating that PE sIL-2R has moderate diagnostic accuracy for TPE.

NLR and PLR are two meaningful measures used for ruling in or out target disease. PLR >10 or NLR <0.1 suggests high accuracy for ruling out or ruling in target disease [40]. The PLR of PE sIL-2R was 10.5 (95% CI: 3.2–34.3), indicating that patients with TPE have about ten times higher chance of a positive PE sIL-2R compared to non-TPE patients. On the other hand, the NLR for PE sIL-2R was 0.20 (95% CI: 0.16–0.27), indicating that non-TPE patients have an approximately five times higher chance of negative PE sIL-2R than TPE patients. Considering that the post-test probability (predictive value) of the index test was affected by both likelihood ratio and the prevalence of target disease, we summarized PPV and NPV of PE sIL-2R at different prevalence. Notably, the NPV of sIL-2R was 98.92% when the prevalence of TPE is 5%, indicating that the patients in low TPE prevalence areas with negative sIL-2R have an extremely low probability of TPE. Therefore, negative PE sIL-2R can be used for ruling TPE in low TPE prevalence areas. While in high TPE prevalence areas, the PPV is only 91.01%. In our opinion, positive sIL-2R cannot be used for confirming TPE under such condition.

In this study, QUADAS-2 was used to evaluate the quality of the included studies. We observed bias in patient selection and index test. Some of the eligible studies did not report whether the participants were consecutively enrolled and did not avoid inappropriate exclusion, which may impair the representativeness of the subjects in eligible studies. Most studies did not use a prespecified cut-off to define positive results, which may overestimate the diagnostic accuracy of PE sIL-2R [18]. Future studies are needed to rigorously evaluate the diagnostic accuracy of sIL-2R.

In addition to pooling sensitivity and specificity, heterogeneity exploration is also an aim of meta-analysis. In this meta-analysis, significant heterogeneity was observed in eligible studies (*I*^2^ = 96%). Therefore, we performed a meta-regression to explore the possible sources of heterogeneity. We found that the type of data collection, reference for TPE diagnosis, country source of study, and sample size were not the sources of heterogeneity. This result may be due to the fact that the number of eligible studies was small (*n* = 9). Further studies are needed to address this issue.

To present, many soluble markers in PE have been proposed to diagnose TPE, which have been summarized in our previous review [9]. Generally, ADA [41], IFN-*γ* [11], and IL-27 [42] represent the most promising diagnostic marker. ADA is an immunosuppressor that can prevent excessive inflammatory response by catalyzing the deamination of adenosine [43]. IFN-*γ* and IL-27 are released by T cells or activated antigen-presenting cells in response to *Mtb* [44, 45]. Compared with these markers, the diagnostic accuracy of sIL-2R is inferior. Under such condition, the clinical implication of PE sIL-2R is limited. In our opinion, it only can be used in the case where ADA, interferon-*γ*, and interleukin-27 cannot be tested.

The present systematic review and meta-analysis has some limitations. The major limitation of this study is the number of eligible study is small. In addition, although we performed a meta-regression to explore the possible sources of heterogeneity but failed to identify the possible sources.

## 5. Conclusion

In conclusion, our results reveal that PE sIL-2R seems to be a useful diagnostic marker for TPE. However, given the improper study design, further rigorous studies are needed to evaluate the diagnostic accuracy of pleural fluid sIL-2R for TPE. sIL-2R shows inferior diagnostic accuracy when compared with the most promising diagnostic markers. Therefore, PE sIL-2R is not preferred for TPE diagnostics at the current stage.

## Figures and Tables

**Table 1:** Summary of eligible studies.

Author	Year	Country	TPE/non-TPE	Non-TPE	Consecutive	Data collection	Target population	sIL-2R assay	Reference standard
Tural [27]	2015	Turkey	52/68	MPE, PPE	No	Unclear	Undiagnosed exudative PE	ELISA	Stain, culture, biopsy
Klimiuk [28]	2015	Poland	44/129	MPE, PPE, TRPE, miscellaneous	Yes	Prospective	Undiagnosed PE	ELISA	Stain, culture, biopsy, treatment response
Ambade [29]	2011	India	48/33	MPE, others	Unclear	Unclear	Undiagnosed PE	ELISA	Stain, culture, biopsy
Harita [30]	2002	Japan	11/39	MPE, PPE, HF	Unclear	Retrospective	Undiagnosed PE	DPC Immulyze	Stain, culture, biopsy, treatment response
Porcel [31]	2000	Spain	23/109	MPE, PPE, TRPE, others	Yes	Prospective	Undiagnosed PE	ELISA	Culture, biopsy, treatment response
Chiang [32]	1994	China	27/66	MPE, PPE, UPE, TRPE	Unclear	Unclear	Undiagnosed PE	ELISA	Stain, culture, biopsy
Chang [33]	1994	China	42/69	MPE, PPE, TRPE	Yes	Prospective	Undiagnosed PE	ELISA	Culture, biopsy
Sarandakou [34]	1991	Greece	13/45	MPE, TRPE	Unclear	Unclear	Undiagnosed PE	ELISA	Culture, biopsy, treatment response
Ito [35]	1990	Japan	10/28	MPE, PPE, TRPE	Unclear	Unclear	Undiagnosed PE	ELISA	Culture, biopsy, treatment response

TPE, tuberculous pleural effusion; MPE, malignant pleural effusion; PPE, parapneumonic pleural effusion; HF, heart failure; TRPE, transudative pleural effusion; UPE, uremic pleural effusion; DPC, Diagnostic Products Corporation.

**Table 2:** Quality assessment of the eligible studies.

Author	Risk of bias				Applicability concerns		
	Patient selection	Index test	Reference standard	Flow and timing	Patient selection	Index test	Reference standard
Tural [27]	High	High	Low	Unclear	High	Low	Low
Klimiuk [28]	Low	Unclear	Low	High	Low	Low	Low
Ambade [29]	Unclear	Unclear	Low	High	Low	Low	Low
Harita [30]	Unclear	Unclear	Low	High	Low	Low	Low
Porcel [31]	Low	Unclear	Low	High	Low	Low	Low
Chiang [32]	Unclear	Unclear	Low	High	Low	Low	Low
Chang [33]	Low	Unclear	Low	Unclear	Low	Low	Low
Sarandakou [34]	High	High	Low	High	Low	Low	Low
Ito [35]	Unclear	High	Low	High	Low	Low	Low

**Table 3:** Diagnostic accuracy of sIL-2R in eligible studies.

Author	AUC	Cutoff	Sensitivity (%)	Specificity (%)	TP	FP	FN	TN
Tural [27]	NR	4800 pg/mL	83	71	43	20	9	48
Klimiuk [28]	0.57	2047.7 pg/ml	79	41	35	77	9	52
Ambade [29]	0.80	4257.4 pg/mL	88	70	42	10	6	23
Harita [30]	NR	2980 U/mL	73	88	8	6	3	43
Porcel [31]	0.96	4700 U/mL	91	95	21	6	2	103
Chiang [32]	NR	5000 U/mL	74	94	20	4	7	62
Chang [33]	NR	4291.4 U/mL	81	100	34	0	8	69
Sarandakou [34]	NR	3777 U/mL	62	98	8	1	5	44
Ito [35]	NR	4500 U/mL	90	100	9	0	1	28

AUC, area under the curve; NR, not reported; TP, true positive; FP, false positive; TN, true negative; FN, false negative.

**Table 4:** Positive and negative predictive values of sIL-2R with different prevalence of TPE in the target population.

Prevalence of TPE (%)	Positive predictive value (%)	Negative predictive value (%)
5	34.76	98.92
10	52.94	97.76
20	71.68	95.09
30	81.27	91.87
50	91.01	82.88
70	95.94	67.48

**Table 5:** Meta-regression analysis.

Parameter	Category	Number of study	Sensitivity		Specificity		*I* ^2^ in joint model	
			Estimates (95% CI)	*P* value	Estimates (95% CI)	*P* value	Estimates (95% CI)	*P* value
Asia	Yes	6	0.82 (0.76–0.88)	0.02	0.94 (0.84–1.00)	0.25	0.00 (0.00–1.00)	0.77
	No	3	0.79 (0.70–0.89)		0.89 (0.68–1.00)			

Sample size >100	Yes	4	0.83 (0.77–0.88)	0.01	0.88 (0.70–1.00)	0.81	0.00 (0.00–1.00)	0.70
	No	5	0.79 (0.72–0.87)		0.94 (0.86–1.00)			

Consecutive enrollment	Yes	3	0.83 (0.75–0.90)	0.01	0.92 (0.77–1.00)	0.50	0.00 (0.00–1.00)	0.90
	No or unknown	6	0.80 (0.74–0.87)		0.92 (0.81–1.00)			

Prospective design	Yes	3	0.83 (0.75–0.90)	0.01	0.92 (0.77–1.00)	0.50	0.00 (0.00–1.00)	0.90
	No or unknown	6	0.80 (0.74–0.87)		0.92 (0.81–1.00)			

Treatment response used	Yes	5	0.80 (0.71–0.88)	<0.01	0.93 (0.81–1.00)	0.49	0.00 (0.00–1.00)	0.88
	No	4	0.82 (0.76–0.88)		0.92 (0.77–1.00)			

## Data Availability

The data supporting the findings of this study are available within the article.

## Abbreviations

TPE: Tuberculous pleural effusion
PE: Pleural effusion
SIL-2R: Soluble interleukin-2 receptor
Mtb: *Mycobacterium tuberculosis*
ADA: Adenosine deaminase
INF-*γ*: Interferon-gamma
IL-27: Interleukin-27
ROC: Receiver operating characteristic
AUC: Area under the receiver operating characteristic curve
TP: True positive
FP: False positive
FN: False negative
TN: True negative
QUADAS-2: revised Quality Assessment for Diagnostic Accuracy Studies
sROC: Summary receiver operating characteristic
MPE: Malignant pleural effusion
PPE: Parapneumonic pleural effusion
TRPE: Transudative pleural effusion
HF: Heart failure
UPE: Uremic pleural effusion
DPC: Diagnostic Products Corporation
PLR: Positive likelihood ratio
NLR: Negative likelihood ratio
DOR: Diagnostic odds ratio
PPV: Positive predictive values
NPV: Negative predictive values.
